# High-Fat Feeding Alters Circulating Triglyceride Composition: Roles of FFA Desaturation and ω-3 Fatty Acid Availability

**DOI:** 10.3390/ijms25168810

**Published:** 2024-08-13

**Authors:** Tong Shen, Youngtaek Oh, Shinwu Jeong, Suengmok Cho, Oliver Fiehn, Jang H. Youn

**Affiliations:** 1 West Coast Metabolomics Center, University of California Davis Genome Center, Davis, CA 95616, USA; tsshen@ucdavis.edu (T.S.); ofiehn@ucdavis.edu (O.F.); 2 Department of Physiology and Neuroscience, Keck School of Medicine, University of Southern California, Los Angeles, CA 90033, USA; youngoh@usc.edu (Y.O.); scho@pknu.ac.kr (S.C.); 3 Department of Ophthalmology, USC Roski Eye Institute, Keck School of Medicine of USC, Los Angeles, CA 90033, USA; shinwuje@med.usc.edu

**Keywords:** lipidomics analysis, palmitoleic acid, fat oxidation, polyunsaturated fatty acids, cardiovascular disease, diabetes

## Abstract

Hypertriglyceridemia is a risk factor for type 2 diabetes and cardiovascular disease (CVD). Plasma triglycerides (TGs) are a key factor for assessing the risk of diabetes or CVD. However, previous lipidomics studies have demonstrated that not all TG molecules behave the same way. Individual TGs with different fatty acid compositions are regulated differentially under various conditions. In addition, distinct groups of TGs were identified to be associated with increased diabetes risk (TGs with lower carbon number [C#] and double-bond number [DB#]), or with decreased risk (TGs with higher C# and DB#). In this study, we examined the effects of high-fat feeding in rats on plasma lipid profiles with special attention to TG profiles. Wistar rats were maintained on either a low-fat (control) or high-fat diet (HFD) for 2 weeks. Plasma samples were obtained before and 2.5 h after a meal (*n* = 10 each) and subjected to lipidomics analyses. High-fat feeding significantly impacted circulating lipid profiles, with the most significant effects observed on TG profile. The effects of an HFD on individual TG species depended on DB# in their fatty acid chains; an HFD increased TGs with low DB#, associated with increased diabetes risk, but decreased TGs with high DB#, associated with decreased risk. These changes in TGs with an HFD were associated with decreased indices of hepatic stearoyl-CoA desaturase (SCD) activity, assessed from hepatic fatty acid profiles. Decreased SCD activity would reduce the conversion of saturated to monounsaturated fatty acids, contributing to the increases in saturated TGs or TGs with low DB#. In addition, an HFD selectively depleted ω-3 polyunsaturated fatty acids (PUFAs), contributing to the decreases in TGs with high DB#. Thus, an HFD had profound impacts on circulating TG profiles. Some of these changes were at least partly explained by decreased hepatic SCD activity and depleted ω-3 PUFA.

## 1. Introduction

Dyslipidemia is a major risk factor for atherosclerotic cardiovascular diseases [1]. Particularly, hypertriglyceridemia is also a known risk factor for type 2 diabetes, and plasma triglyceride (TG) levels have been considered a major component in the assessment of diabetes or CVD risks [2]. However, recent lipidomics studies have demonstrated that not all TG molecules behave the same way; individual TG species are regulated differently under various metabolic conditions [2,3,4]. TGs are composed of three acyl chains, each varying in carbon number and double-bond content. The combinations of three different acyl chains produce many different species of unique TGs, which can be individually measured by lipidomics that detail more than 100 different TG species from plasma samples [3]. Hence, studying differential regulation of individual TG molecules by lipidomics can provide insights into underlying mechanisms and associations with metabolic or cardiovascular risks.

Rhee et al. demonstrated that TGs with different fatty acid compositions responded differentially to insulin action or insulin resistance [2]. They also reported that distinct TG groups were associated with either an increased or decreased diabetes risk; TGs of lower carbon number and double-bond content were associated with increased diabetes risk, whereas TGs of higher carbon number and double-bond content were associated with decreased diabetes risk [2]. We previously identified two distinct TG groups in the blood, differentially responding to antibiotic treatment in rats [3]. Most detected TG species were downregulated, whereas a smaller group was upregulated by antibiotic treatment. Interestingly, these upregulated TGs comprised long-chain PUFAs [2]. Furthermore, the same TG group was downregulated by a high-sodium and low-potassium (HNaLK) diet, suggesting the existence of mechanisms under certain diet conditions that differentially regulate this group of long-chain PUFA-based TGs and, thus, diabetes risk.

The mechanisms underlying the differential TG regulations are unclear. One goal of the present study was to examine the effects of an HFD on circulating TG profiles in rats and test the hypothesis that an HFD reduces TGs composed of long-chain PUFAs, as previously seen in rats maintained on a HNaLK diet [3]. Thus, we tested if common changes in TG profiles are observed with the two diets (i.e., HFD and HNaLK diets) associated with increased diabetes and/or CVD risks [5,6,7]. A second goal of the study was to examine the effects of acute feeding (a single meal) on circulating lipid profiles in control and high-fat-fed rats. Acute feeding is expected to impact circulating lipid profiles because diets directly impact chylomicron-derived lipids. In addition, the acute feeding response may be altered by an HFD to account for some of the chronic effects of an HFD. For these reasons we performed lipidomics analysis on plasma samples collected both in the pre- and postprandial states in the control and HFD groups. Lipidomics analysis was combined with general metabolomics analysis, which provides measurements of saturated free fatty acids (FFAs) and other metabolic indicators, such as glycerol (as lipolysis indicator) or 3-hydroxybutyrate (ketosis indicator), that were not measured in our LC-MS-based lipidomics assay. Animals were fed an HFD only for 2 weeks; this short-term high-fat feeding was employed to avoid secondary responses to changes in body weight or body compositions that occur after prolonged high-fat feeding. The results provided novel insights into the differential behaviors of individual TGs and identified specific changes (i.e., decreased indices of stearoyl-CoA desaturase [SCD] activity and depletion of ω-3 FFAs) associated with short-term high-fat feeding.

## 2. Results

### 2.1. Effects of Diet on Energy Intake, Weight Gain, and Metabolic Parameters

**Figure 1:**
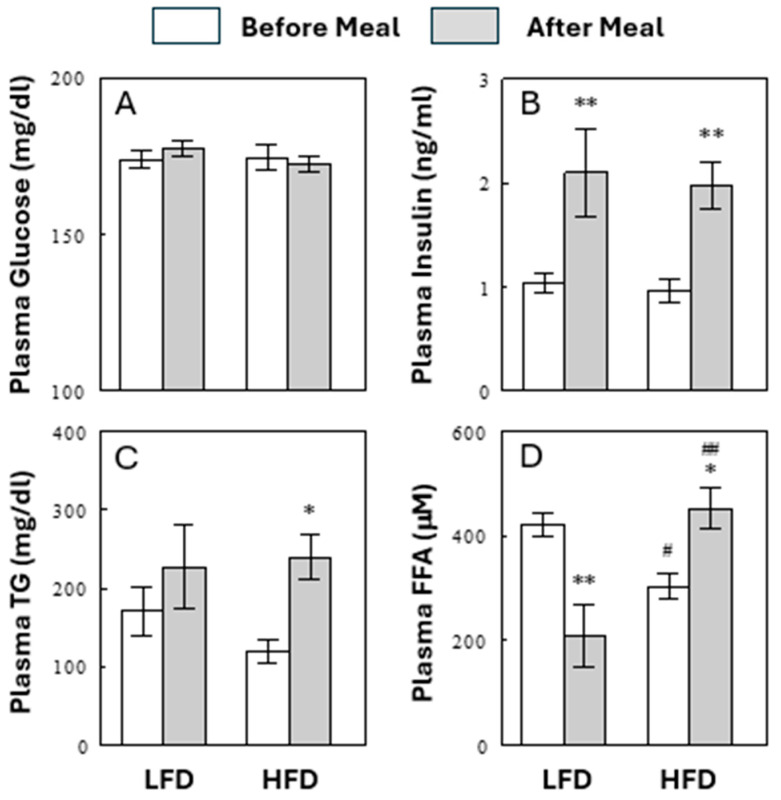
Effects of diets on plasma glucose (**A**), insulin (**B**), TG (**C**), and FFA (**D**) in the preprandial (Before Meal) and postprandial (After Meal) states. Data are means ± SEM (*n* = 10). *, *p* < 0.05; **, *p* < 0.01 vs. Before Meal; #, *p* < 0.05; ##, *p* < 0.01 vs. LFD.

Animals were maintained on either a low- (10%, LFD, control) or high- (60%, HFD) fat diet for 2 weeks. The diet compositions are shown in Table 1. Calorie intake was 15% higher with the HFD than the LFD in the first week (*p* < 0.001) but not in the second week (Table 1). Initial and final weights and weight gain were not significantly different between the two diet groups. In each diet group, blood samples were obtained before (“Before Meal”) or 2.5 h after (“After Meal”) the start of normal feeding at 6 PM, and plasma samples were analyzed for metabolic parameters. Plasma glucose and insulin levels were not altered by the HFD either before or after a meal (Figure 1A,B). The insulin resistance index HOMA-IR was not different between the two diet groups (12.9 ± 1.3 for the LFD group vs. 12.2 ± 1.5 for the HFD group, *p* > 0.05). In contrast, plasma total triglyceride (TG) levels increased significantly after a meal with an HFD (*p* < 0.05) but not with an LFD (Figure 1C). Plasma total FFA levels decreased after a meal with a LFD but increased with an HFD, resulting in higher after-meal FFAs in the HFD than in the LFD group (*p* < 0.001; Figure 1D). Thus, when rats were fed for a short term (i.e., 2 weeks) with an HFD, we observed significant alterations in before- and/or after-meal total levels of FFA and TG, but not in energy intake, body weight, plasma glucose and insulin levels, or insulin resistance index.

### 2.2. Effects of Diet and Meal on Plasma Lipid Profiles

**Figure 2:**
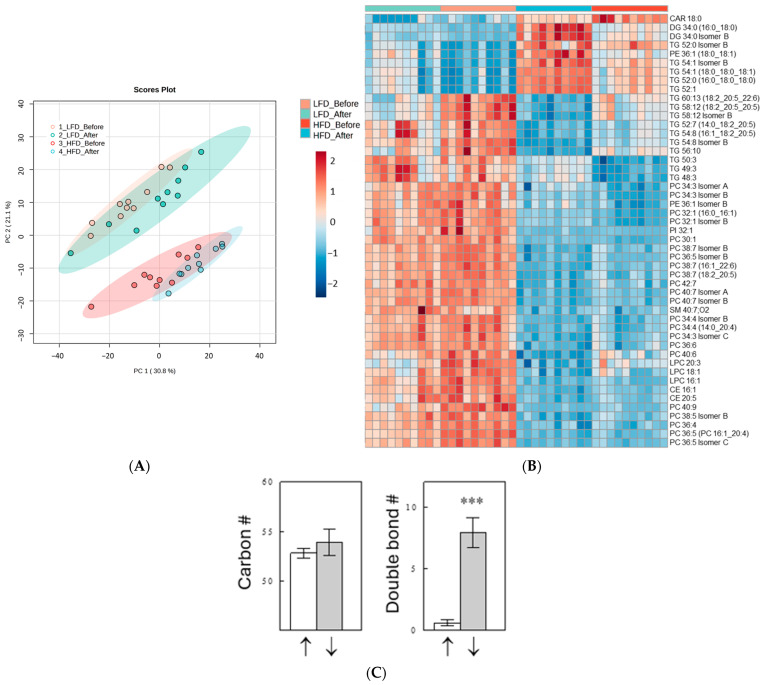
Unsupervised PCA plot of plasma lipidomes (**A**); heat map showing the top 50 most significantly changed lipids in plasma among the four groups (**B**); carbon and double-bond numbers of TGs, included in the heat map, significantly increased (“↑”) or decreased (“↓”) by the HFD (**C**). In (**C**), data are means ± SEM (*n* [the number of TG species] = 5 for ↑ and 7 for ↓). ***, *p* < 0.001 vs. I.

Lipidomics analyses annotated structures of 743 lipids, including acylcarnitines (*n* = 6), cholesterol esters (*n* = 14), ceramides (*n* = 50), diacylglycerides (*n* = 23), FFAs (*n* = 25), galactosylceramides (*n* = 7), lysophosphatidylcholines (*n* = 44), lysophosphatidylethanolamines (*n* = 6), phosphatidylcholines (PCs; *n* = 158), phosphatidylethanolamines (PEs; *n* = 49), phosphatidylinositols (*n* = 25), sphingomyelins (*n* = 64), and TG species (*n* = 214). An unsupervised principal component analysis (PCA) showed that the main separation in the plasma lipidome was driven by diet (i.e., HFD vs. LFD) not by meal time (i.e., Before vs. After Meal; Figure 2A), although the meal changed the plasma lipidome within each diet group. The Kruskal–Wallis one-way ANOVA (adjusted for false detection rates) detected 529 of 733 lipids significantly changed by diet and/or meal. Figure 2B shows the top 50 lipids with the most significant changes in a heat map. Fifteen of the top twenty most significant lipid changes were found in the class of TGs. Interestingly, these TGs showed two distinct patterns of changes: one group of TGs (*n* = 10) was downregulated, whereas the other group (*n* = 5) was upregulated by the HFD. Figure 2C shows that the two TG groups were distinguished mainly by the number of double-bonds in their acyl chains (i.e., fatty acids). TGs composed of highly unsaturated fatty acids (average double-bond number [DB#] = 7.9) were downregulated, whereas those mainly composed with saturated or monounsaturated fatty acids (average DB# = 0.6) were upregulated by the HFD. Similar results were obtained when all TGs were included in the analysis that were significantly altered by more than twofold by the HFD under both before- and after-meal conditions, *n* = 22 for downregulated TGs and 10 for upregulated TGs (Supplemental Figure S1).

### 2.3. Effects of a Meal on Plasma Lipid Profiles

**Figure 3:**
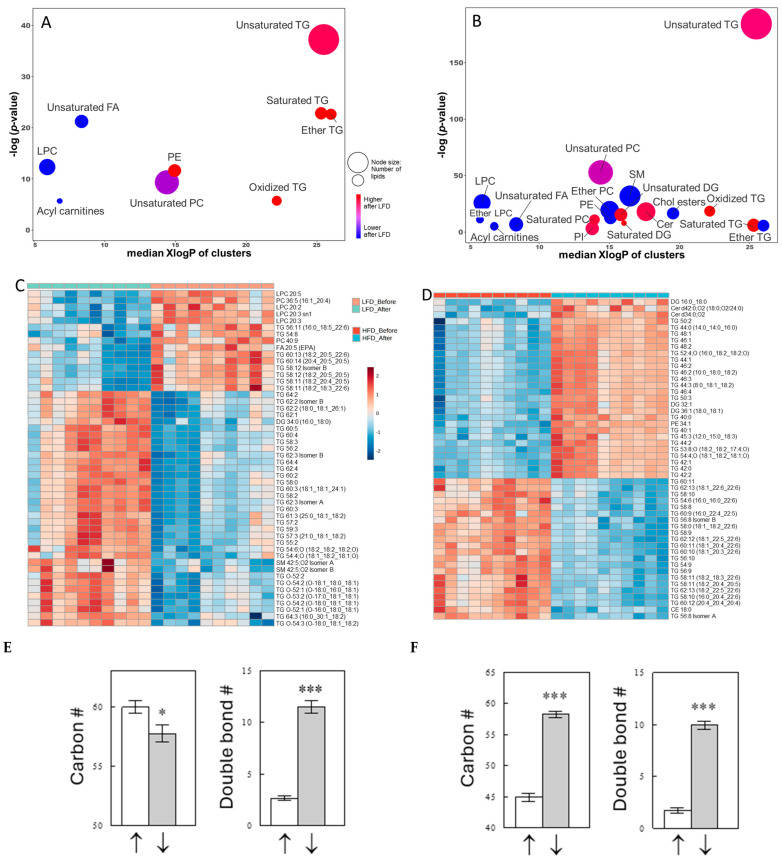
ChemRICH plots for acute feeding effects with an LFD (**A**) or HFD (**B**) and heat maps showing the top 50 lipids in plasma most significantly altered by acute feeding with an LFD (**C**) or HFD (**D**). Also shown are carbon and double-bond numbers of TGs, included in the heat maps, significantly increased (“↑”) or decreased (“↓”) with the LFD (**E**) or HFD (**F**). Data are means ± SEM (*n* [the number of TG species] = 8 for ↑ and 22 for ↓ in (**E**); *n* = 24 for ↑ and 21 for ↓ in (**F**)). *, *p* < 0.05; ***, *p* < 0.001 vs. ↑. The table form of this ChemRICH output can be found in Supplemental Table S1.

We next examined the effect of a meal on lipid metabolites in each diet group. ChemRICH analysis identified several lipid clusters that were significantly altered by the LFD or HFD meal (Figure 3A,B; metabolites within each cluster can be found in Supplemental Table S1). The most impressive effects of a meal in both diet groups were seen with unsaturated TGs. The top 50 lipids with the most significant changes are shown in a heat map (Figure 3C,D), and most of these lipids are TGs. This is not surprising considering that TGs are absorbed from the diets via chylomicrons, and rises in TG levels are expected after a meal. Interestingly, some TGs were downregulated, whereas other TGs were upregulated in both diet groups. In the LFD group, the two TG groups differentially regulated by a meal were distinguished mainly by the acyl chain double-bond content (Figure 3E). In contrast, in the HFD group, the two groups differentially regulated by a meal differed in both their fatty acid carbon and double-bond numbers (Figure 3F). Maintaining the HFD caused decreases in the levels of TGs composed of highly unsaturated long-chain fatty acids but increases in TGs mainly composed of saturated or monounsaturated short-chain fatty acids.

LFD meals also significantly affected unsaturated FFAs; 20 out of 22 unsaturated FFAs were downregulated after a meal, as indicated by the blue color in the ChemRICH plot (Figure 3A), and 10 FFAs were significant at *p* < 0.05. This downregulation of plasma FFA levels is likely due to suppression of lipolysis via increasing insulin levels (Figure 1B). Interestingly, in the HFD group, only five unsaturated FFAs were found significantly downregulated at *p* < 0.05 after a meal (Figure 3B), suggesting that the insulin effects to suppress lipolysis were impaired in the HFD group. In addition, meals in the LFD group also significantly increased saturated and ether TGs (O-TGs) (Figure 3A), and again, these acute postprandial effects were less significant in the HFD group (Figure 3B), suggesting such regulations were impaired by long-term HFD treatments.

### 2.4. Effects of Diet and Meal on Plasma TG Profiles

**Figure 4:**
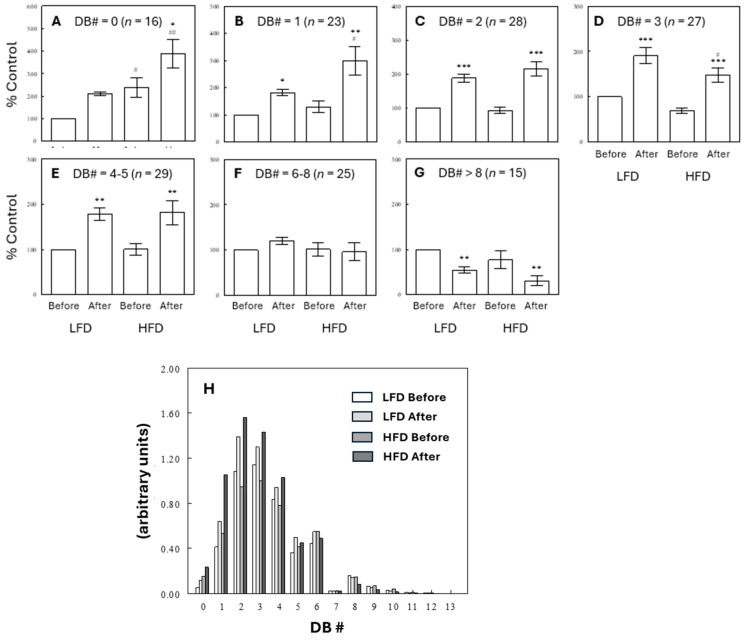
Effects of diets and meal on plasma TGs with different double-bond numbers (DB#), expressed as % control (i.e., before meal in the LFD group) (**A**–**G**) or as absolute levels (**H**). Data are means ± SEM (for *n*, the number of different TG species). *, *p* < 0.05; **, *p* < 0.01; ***, *p* < 0.001 vs. Before Meal; #, *p* < 0.05; ##, *p* < 0.01 vs. LFD. In (**H**), symbols for statistical significance are omitted for simplicity.

Figure 4 shows patterns of changes in TGs with different double-bond numbers (DB#s) under different diet and meal conditions. First, unsaturated TGs with 2–5 DBs increased after a meal, but these effects were not generally altered in rats maintained on an HFD (Figure 4C–E). These after-meal increases may be due to diet-derived TGs in chylomicrons containing fatty acids with a DB# of 1 (C18:1, oleate) or 2 (C18:2, linoleate) that are abundant in the diets. Second, saturated TGs with 0–1 DBs increased after a meal more under HFD than LFD regimes (Figure 4A,B). These meal effects are likely due to the absorption of saturated and monosaturated FFAs directly derived from the diets, in addition to de novo synthesis of fatty acids after a meal under the effect of insulin. The larger postprandial effects of HFD meals than LFD meals may reflect higher contents of saturated and monosaturated fatty acyl groups in the HFD than in the LFD. In contrast, TGs with >5 DBs did not increase after meals (Figure 4F,G). Interestingly, polyunsaturated TGs with >8 DBs decreased after a meal, and these meal effects showed a tendency to be larger under HFD than LFD conditions (Figure 4G). Thus, TG species exhibited different behaviors depending on the number of double-bonds, and these patterns of changes are consistent with the overall HFD to LFD comparisons where TG levels with low DB#s increased but TG levels with high DB#s decreased under HFD treatments (Figure 2C). Figure 4H shows relative contributions of TGs with different DB contents to the total TG mass in the four experimental groups. The major TG groups in terms of mass were those with DB#s of 2–4, which were not significantly affected by the HFD. TGs containing highly polyunsaturated fatty acids (e.g., DB#s > 8) may not contribute much to the total TG mass (as energy source), estimated to account for 1–3% of total TG mass. However, PUFA-containing TGs are important precursors for oxylipins, lipid mediators that serve as regulators of various physiological functions.

### 2.5. Decreased SCD Activity Indices in the HFD Group

**Figure 5:**
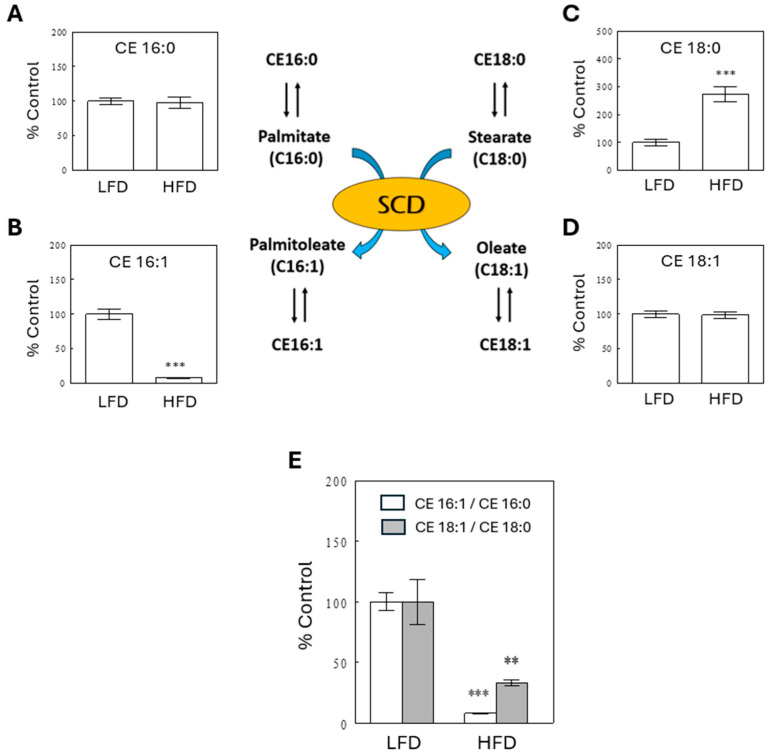
A schematic diagram illustrating the function of SCD to convert saturated fatty acids (palmitate and stearate) to monounsaturated fatty acids (palmitoleate and oleate) and rapid interconversions between CEs and FFAs in the liver. Hepatic SCD activity (**E**) was estimated from the ratios of CE 16:1 (**B**) to CE 16:0 (**A**) or CE 18:1 (**D**) to CE 18:0 (**C**). Data are means ± SEM (*n* = 10). **, *p* < 0.01; ***, *p* < 0.001 vs. LFD.

To understand the differential effects of an HFD on TGs with different DB#s, we next examined changes in plasma FFAs, which are building blocks for hepatic TG synthesis. The most significant changes among circulating FFAs were observed with palmitoleic acid (C16:1), which is produced from palmitate (C16:0) by stearoyl-CoA desaturase (SCD). SCD is an enzyme that catalyzes a rate-limiting step in the synthesis of unsaturated fatty acids. SCD also catalyzes the conversion of stearic acid (C18:0) to oleate (C18:1). SCD activity has been inferred by assessing the product to substrate ratios (i.e., C16:1/C16:0 or C18:1/C18:0; Figure 5) [8]. Our lipidomics assay, based on LC-MS, does not report saturated FFAs due to high background levels. However, it detects cholesterol esters (CEs), formed by the esterification of cholesterol with fatty acids in the liver, including saturated fatty acids. These fatty acid esters may reflect FFA levels in the liver. Using CE data, we estimated SCD activity as the ratios of CE 16:1 to CE 16:0 and of CE 18:1 to CE 18:0. Figure 5 shows that these ratios significantly decreased in the HFD than in the LFD group when compared in the basal states (i.e., before meal). These data indicate that SCD activity profoundly decreased in the HFD group, which might cause impaired conversion of saturated to unsaturated fatty acids, and this may explain, at least in part, why saturated TGs with a 0–1 DB# increased in the HFD group (Figure 4A,B).

### 2.6. Depletion of Circulating ω-3 FFAs by HFD

In addition to palmitoleic acid (C16:1), the following FFAs were significantly decreased in plasma under chronic HFD conditions (before meal): eicosapentaenoic acid (EPA, C20:5), docosahexaenoic acid (DHA, 22:6), γ-linolenic acid (C18:3), and physeteric acid (C14:1) (*p* < 0.05 after adjustment for multiple comparisons; Table 2). Of these FFAs, EPA and DHA are ω-3 FFAs that are found abundantly in fish and some plant oils. Another major ω-3 FFA α-linoleic acid (C18:3) decreased significantly in the HFD group (*p* < 0.01), as reported by the GC-TOF MS assay. Thus, all major ω-3 FFAs detected in plasma decreased in rats maintained on the HFD. In contrast, ω-6 FFAs, except γ-linolenic acid (C18:3), were not significantly altered by the HFD (*p* > 0.05). These data suggest that ω-3 FFAs were selectively depleted in rats maintained on the HFD. The depletion of the circulating ω-3 FFAs, which are highly unsaturated, may explain, at least in part, decreases in TGs with high DB#s in the HFD group (Figure 2C).

### 2.7. Effects of Diet and Meal on Oxidized TGs and Ether TGs

**Figure 6:**
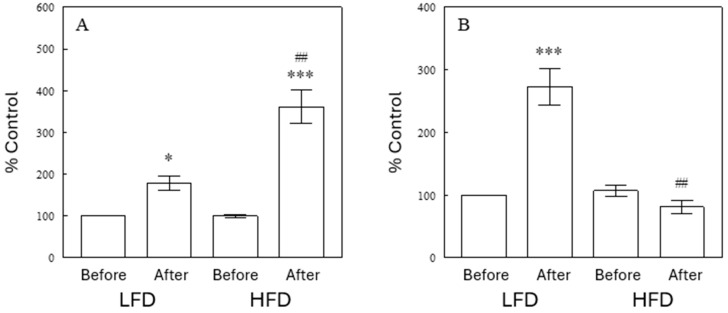
Effects of diets on circulating oxidized TGs (**A**) and ether TGs (**B**) in the preprandial (Before Meal) and postprandial (After Meal) states. Data are means ± SEM (*n* = 10). *, *p* < 0.05; ***, *p* < 0.001 vs. Before Meal; ##, *p* < 0.001 vs. LFD.

The lipidomics assay detected several TGs containing oxidized fatty acids. Such partly oxidized TGs increased after a meal under LFD conditions (*p* < 0.05, *n* = 11), but this effect was considerably larger after a meal with the HFD (*p* < 0.001 vs. LFD; Figure 6A). Oxidized TGs may reflect oxidative stress, and these data may indicate increased postprandial oxidative stress in the HFD group. Interestingly, this pattern was not seen with other plasma lipids with partially oxidized fatty acyls, such as ceramides (*n* = 14), FFAs (*n* = 2), SMs (*n* = 37), and STs (*n* = 4) (see Supplemental Figure S2). This finding suggests that the oxidative stress effect may be specific to TGs, possibly due to oxidation of FFAs in the process of intestinal lipolysis, transport across the intestinal mucosa, and re-esterification into TGs before uptake by chylomicrons. Hence, these data may indicate an increase in intestinal oxidative stress under HFD due to increased fat contents, compared to an LFD. In addition, 12 TG species were identified to contain ether bonds. Such ether TGs also showed increased levels after a meal under LFD conditions, but not in the HFD group (Figure 6B). These data suggest that the production of ether lipids in peroxisomes may be increased after a meal, but that this process is impaired in the HFD group. However, ether PC or ether PE lipids showed very minimal changes after a meal, supporting a low turnover of these structural lipids in comparison to TG lipids (Supplemental Figure S3).

### 2.8. Lipids and Markers of Lipid Metabolism Detected by GC-TOF MS

**Figure 7:**
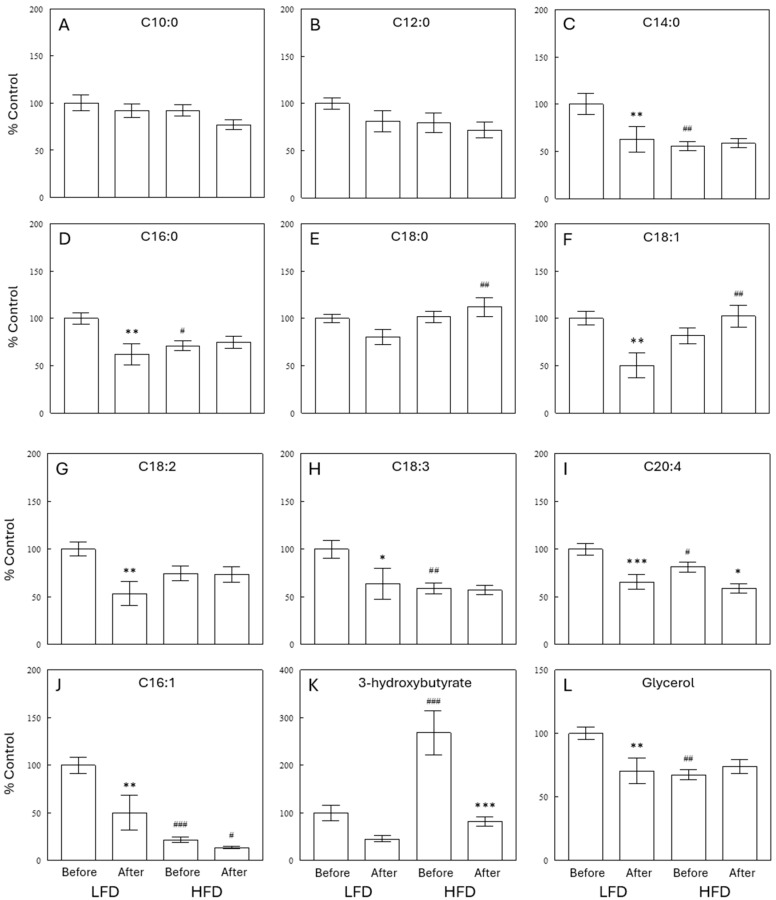
Effects of diets on circulating levels of various FFAs (**A**–**J**), 3-hydroxybutyrate (**K**), and glycerol (**L**) in the preprandial (Before Meal) and postprandial (After Meal) states, measured using a GC-TOF MS analysis. Data are means ± SEM (*n* = 10). *, *p* < 0.05; **, *p* < 0.01; ***, *p* < 0.001 vs. Before Meal; #, *p* < 0.05; ##, *p* < 0.01; ###, *p* < 0.001 vs. LFD.

The gas chromatography–time of flight mass spectrometry (GC-TOF MS) assay detected 154 known primary metabolites, including saturated FFAs and markers of lipid metabolism. Some of these were not reliably detected by the lipidomics assay. Figure 7 shows that medium chain FFAs (C10:0 and C12:0) were not different among the four experimental groups (Figure 7A,B). In contrast, long-chain FFAs, both saturated (C14:0, C16:0, and C18:0) and unsaturated (C18:1, C18:2, and C18:3), showed similar postprandial decreases after LFD meals, but not after HFD meals (Figure 7C–H). These patterns were parallel to changes in plasma glycerol (Figure 7L), a lipolysis marker. This finding supports the interpretation that lipolysis was inhibited after a meal with a LFD, but less so in the HFD group. Interestingly, arachidonic acid (C20:4) decreased after a meal in both diet groups (Figure 7I). The profound effect of the HFD to decrease palmitoleic acid (C16:1), detected by the lipidomics assay (Figure 6), was confirmed by the GC-TOF MS assay (Figure 7J). In addition, GC-TOF MS data confirmed significant effects of the HFD to decrease SCD activity indices, calculated from plasma FFAs as the ratios of C16:1 to C16:0 and of C18:1 to C18:0 (Supplemental Figure S4). Plasma levels of ω-3 FFA α-linoleic acid (C18:3) were decreased by the HFD (basal states; *p* < 0.01). Finally, plasma levels of 3-hydroxybutyrate increased substantially in the basal state of the HFD group (Figure 7K), reflecting increased fatty acid oxidation (see Section 3).

## 3. Discussion

The present study demonstrates that high-fat feeding had significant impacts on circulating lipid profiles with most significant effects observed on TG profiles. Importantly, the effect of an HFD on individual TG species was different depending on the number of double-bonds in their fatty acid chains. A high-fat diet increased TGs with low DB# but decreased those with high DB#. This data adds evidence for the hypothesis of differential regulation of individual TG species under various metabolic conditions [2,3,4]. Some of these changes may be explained by HFD-induced changes in circulating and hepatic FFAs, building blocks for hepatic TG synthesis, which revealed that an HFD decreased hepatic SCD activity indices and depleted ω-3 PUFAs. Decreased SCD activity would reduce the conversion of saturated (dietary or de novo synthesized) to monounsaturated FFAs (MUFAs), contributing to the increases in saturated TGs or TGs with low DB# with an HFD, observed both in pre- and postprandial states (Figure 4). On the other hand, depleted ω-3 PUFAs appear to contribute to the decreases in TGs with high DB#. Thus, altered SCD activity and ω-3 PUFA availability explain, at least in part, the differential behaviors of circulating TGs with high vs. low DB# in response to an HFD.

The HFD effects to increase TGs with low DB# and decrease TGs with high DB# are similar to those observed in our previous study with a HNaLK diet [3]. High fat and HNaLK are two major features of Western diets, known to increase risks of type 2 diabetes and CVD. TGs with long-chain PUFAs were shown to be associated with decreased risks of diabetes, in opposite to TGs with low DB# that increase diabetes risks [2]. Taken together, the common changes observed with the HFD and HNaLK diets may be related to diabetes and cardiovascular risks associated with these diets. Although similar patterns were observed with the two diets in the regulation of distinct TG groups, ω-3 PUFA depletion or decreased SCD activity indices were observed with the HFD (present data) but not with the HNaLK diet, suggesting the underlying mechanisms may be different. If so, it would be important to test if the effects of an HFD and HNaLK diet on TG profiles (and on diabetes and cardiovascular risks) are additive.

The present data demonstrate that TGs containing PUFAs may represent only a small fraction (e.g., 1–3% for TGs with DB# > 8) of the total TG pool (Figure 4). However, these TGs may be important as the major sources of circulating PUFAs, which are precursors of oxylipins, many of which serve as regulators of cell functions. For example, increased consumption of EPA (C20:5) prevents insulin resistance and impaired glucose homeostasis, and substantial evidence exists that hydroxyeicosapentaenoic acids (HEPEs), derived from the EPA by lipoxygenase, may be a major mediator of those beneficial effects of EPA [9]. Also, epoxyeicosatrienoic acids (EETs), produced from arachidonic acid (C20:4) by cytochrome P450, play a role in the regulation of endothelial function [10]. Depletion of PUFAs may lead to a depletion of these oxylipins to cause impairments of insulin action and endothelial function, major risk factors for type 2 diabetes and CVD. Hence, understanding the regulation of highly unsaturated TGs under various pathophysiological conditions and its relationship with metabolic and cardiovascular risks is of utmost importance.

Selective depletion of ω-3 PUFAs was also observed in previous studies in high-fat-fed rodents [11,12]. ω-3 PUFAs exert beneficial effects on metabolic control and insulin action, whereas depletion of ω-3 PUFAs contributes to the development of insulin resistance and inflammation with HFDs [9,13,14]. In the present study, dietary content of ω-3 PUFAs was greater in the HFD than in the LFD (see Supplemental Table S2). Therefore, the depletion of ω-3 PUFAs might be due to increased fat oxidation in the HFD group, indicated by increased 3-hydroxybutyrate, a marker for fat oxidation [15]. In contrast, ω-6 (except γ-linolenic acid) or other FFAs were not depleted despite increased fat oxidation because intake of these FFAs was substantially increased due to high fat contents of the diet. Lard, the major fat source in the HFD employed in the present study, contains large quantities of saturated and ω-6 FFAs, but not ω-3 FFAs. Therefore, it seems the high-fat feeding depleted circulating ω-3 FFAs due to increased fat oxidation, which was not fully compensated for by increased dietary intake because of relatively low contents of ω-3 FFAs in our HFD. This effect may not occur if HFDs employed contain substantial amounts of fish or flaxseed oil to provide ω-3 FFAs.

SCD is an enzyme that catalyzes a rate-limiting step in the synthesis of MUFAs from dietary or de novo synthesized saturated fatty acids (SFAs). In the present study, SCD activity was inferred by assessing its product to substrate ratios (i.e., palmitoleate [C16:1] to palmitate [C16:0] or oleate [C18:1] to stearate [C18:0]) [8] using CE data. Circulating CEs are formed by the esterification of cholesterol with FFAs in the liver, and their fatty acid esters reflect FFA levels in the liver. The results indicate that high-fat feeding profoundly decreased indices of SCD activity in the liver (Figure 5). SCD activity indices were also calculated from plasma FFAs, measured by GC-TOF MS. These data confirm significant effects of the HFD to decrease SCD activity indices, with more profound effects on C16:1 to C16:0 ratios than C18:1 to C18:0 ratios (Supplemental Figure S4). The C16:1 to C16:0 ratio may better reflect SCD activity than the C18:1 to C18:0 ratio because the latter may be biased by high dietary intake of C18:1 in the HFD group. The ratio of C16:1 to C16:0 was higher in the high-fat than low-fat diet (see Supplemental Table S2). Therefore, the dietary fatty acid composition cannot explain the decrease in this ratio with high-fat feeding. Moreover, the dietary content of palmitoleate (C16:1) was far greater in the HFD than LFD, and, therefore, the quantity of fatty acids supplied from the diet cannot explain the significant decrease in plasma levels of C16:1 in the HFD group. 

Previous studies reported increased [16] or decreased [17,18] hepatic SCD expression or activity with high-fat feeding. This variation may be due to differences in fat composition in the HFDs used. SCD activity and expression increase in response to SFAs but decrease after ingestion of MUFAs or PUFAs [19]. The HFD effects on SCD activity and expression may also depend on the duration of high-fat feeding. SCD1 knockout mice are protected against diet-induced obesity [20] and fatty liver disease [21], possibly due to decreased de novo lipogenesis [21,22]. SCD1 knockout mice also exhibit increased plasma levels of ketone bodies [23], suggesting increased fat oxidation. In the present study, decreased SCD activity indices with the HFD were associated with increased plasma levels of the ketone body 3-hydroxybutyrate. Thus, the HFD-induced decrease in SCD activity may serve as a mechanism for protection against diet-induced obesity by inhibiting de novo lipogenesis and increasing fat oxidation, as observed in SCD1 knockout mice [20,21,22]. This protective mechanism may operate at least during an initial period of high-fat feeding, consistent with no significant effects on weight gain during the 2-week high-fat feeding in the present study.

Decreased SCD activity with an HFD resulted in a profound decrease in plasma levels of palmitoleate (C16:1). Palmitoleate was proposed as an adipose tissue-derived lipid hormone that increases insulin action and inhibits hepatosteatosis [24]. Circulating palmitoleate strongly correlated with insulin sensitivity in subjects at increased risk for type 2 diabetes [25], suggesting a major role of palmitoleate in the pathogenesis of insulin resistance in humans. In contrast, insulin resistance in obese people was not associated with decreased palmitoleate in plasma and lipoproteins [26]. In the present study, a dramatic decrease in circulating palmitoleate with the HFD was associated with no significant changes in plasma glucose, insulin, or insulin resistance index. Although these data may argue against a major role of circulating palmitoleate in glucose homeostasis within a physiological (vs. pharmacological in some other studies) range, this important issue warrants further studies designed to directly address it.

Our lipidomics analysis identified specific subclasses of TGs showing distinct patterns of changes with an HFD. One subclass was TGs with oxidized fatty acid chains (ox-TGs), which increased after feeding but more profoundly in the HFD than the control group (Figure 6). Ox-TGs may reflect oxidative stress, and these data may indicate that postprandial oxidative stress is increased in the HFD group [27,28]. Interestingly, this pattern was not seen with other plasma lipids that contain oxidized fatty acid chains, such as ceramides, FFAs, SMs, and STs (see Supplemental Figure S2). Hence, the oxidative stress effect may be specific to TGs, possibly due to oxidized FFAs in the diet, which may be higher with the HFD because of higher fat contents than the control LFD. Alternatively, increased ox-TGs may be due to increased FFA oxidation in the intestine during their absorption and re-esterification into TGs, suggesting oxidative stress in the intestine with the HFD.

Another subclass of TGs was ether analogues of triglycerides (ether TGs; 1-alkyldiacylglycerols). Ether TGs increased substantially after feeding in the control, but not in the HFD group (Figure 6). Ether lipids are lipids with the lipid “tail” group attached to the glycerol backbone via an ether bond that is produced in peroxisomes, instead of the ester linkage found in conventional TGs and glycerophospholipids. Ether lipids, in particular ether glycerophospholipids, affect cell membrane properties, dynamics, and functions, as they are major parts of cell membranes in mammals [29]. They are also known to serve as cell-signaling molecules and act as antioxidants [29,30,31]. Decreased ether lipids, in particular alkenyl phospholipids (i.e., plasmalogens), are associated with various pathologies, such as neurodegenerative diseases, metabolic disorders, and cancer [31]. The precise functions of alkyl ether TGs and their metabolites have been poorly addressed. Ether TGs are byproducts of ether lipid biosynthesis [32], and our data suggest that ether lipid synthesis may be increased after feeding with the control LFD, but this response is impaired with the HFD.

It is unknown whether the changes in ox-TGs and ether-TGs are causally related to or independent of each other. If a causal relationship is established, it would provide novel insights into the mechanisms of regulating these important cellular processes. Synthesis of ether lipids begins in the peroxisome, which contains multiple enzymes to produce 1-O-alkyl-DHAP from DHAP and fatty alcohol. 1-O-alkyl-DHAP is then transported into the endoplasmic reticulum (ER) where various ether lipids form. HFD feeding was shown to induce oxidative stress [33] and ER stress [34]. It would be important to test if an HFD triggers oxidative and/or ER stress in the enterocytes to impair postprandial ether lipid synthesis. Alternatively, it is also conceivable that, in light of the antioxidant roles of ether lipids [30,31], impaired ether lipid production may be linked to increased postprandial oxidative stress in the enterocytes.

## 4. Materials and Methods

### 4.1. Animal Experiments

Wistar rats (male, 200–225 g) were obtained from Envigo Laboratories (Indianapolis, IN, USA). Animals were housed under controlled temperature (22 ± 2 °C) and lighting (12 h light, 6 AM–6 PM; 12 h dark, 6 PM–6 AM) with free access to water and food. The animals were fed a high- (60%) fat diet (Envigo TD.06414) or a low- (10%) fat diet (Envigo TD.08806) ad libitum for 2 weeks (*n* = 20 each, a total of 40 animals). After the 2-week feeding, food was removed from animal cages at 1 PM, and blood samples were obtained immediately before (Before Meal) or 2.5 h after (After Meal; *n* = 10 each) the normal initiation of feeding at 6 PM when lights are off. For this, animals were anesthetized with isoflurane, and blood samples were collected through cardiac puncture. Blood samples were rapidly spun, and plasmas were isolated and stored at −80 °C until analysis. All animal procedures were approved by the USC IACUC.

### 4.2. Chromatographic and Mass Spectrometric Conditions for Lipidomics Analysis

Plasma samples (*n* = 10 for each group; a total of 40 samples) were analyzed by LC-MS/MS with previous method with modification [35]. Briefly, lipids were extracted from 20 µL of plasma using a biphasic extraction with the Matyash method [36]. Method blanks (20 µL of water) were extracted and analyzed alongside samples. A quality control (QC) sample was created by pooling aliquots from each sample. The organic phase was dried down and reconstituted in 0.11 mL methanol/toluene (9:1, *v*/*v*) containing internal standards. The internal standard mix contained UltimateSPLASH ONE (Avanti Polar Lipids, Alabaster, AL, USA) and 3 extra classes (FFAs, acyl carnitines, and cholesterol), which covered 76 deuterium-labeled lipid species across 18 lipid classes. Non-polar metabolites were analyzed on Vanquish UHPLC coupled to a Q-Exactive HF mass spectrometer (Thermo Fisher Scientific, Waltham, MA, USA). Liquid chromatography used a Waters Acquity Premier UPLC BEH C18 column (50 mm × 2.1 mm, 1.7 μm particle size) with VanGuard FIT (Waters, Milford, MA, USA), basically as previously described [3]. Data were collected in Top2-DDA-MS/MS with a scan range from 120 to 1700 *m*/*z*. Samples were analyzed in a randomized order with method blank and pooled quality control samples injected every ten samples. More MS/MS spectra were acquired by repetitively injecting the pool QC with iterative exclusion (IE-Omics) methods [37].

### 4.3. LC-MS Data Processing Using MS-DIAL

Data were processed using open-source software MS-DIAL version 4.92 [38]. Raw data are available on the Metabolomics Workbench (https://metabolomicsworkbench.org/; Project doi: http://dx.doi.org/10.21228/M88N7K, accessed 25 July 2024). MS-DIAL performed peak detection, peak alignment, MS2 spectral deconvolution, adduct identification, blank subtraction, gap filling, and annotation. Lipids were annotated by MS/MS library matching and/or accurate mass/retention time (*m*/*z*-RT) library matching with manual inspection. MS-DIAL parameters were set to 10 scans for minimum peak width, smoothing level of 3, intensity of 10,000 for minimum peak height, MS1 tolerance of 5 mDa, MS2 tolerance of 10 mDa, and retention time window of 0.1 min for retention time matching to an in-house *m*/*z*-RT library. Tandem MS (MS2) spectra were matched to built-in lipid atlas library with at least 70% averaged score [38]. Features were kept after a blank subtraction and with a technical variance lower than 30% in the pooled QC. Mass Spectral Feature List Optimizer performed duplicate peak removal, isotope removal, and combined adducts [39]. Peak height was reported as intensity for each annotated metabolite in each sample, which were comparable between samples and groups. The median relative standard deviation (RSD) of all internal standards was 6%. Thus, no normalization was performed.

### 4.4. Chemical Enrichment Analysis

Chemical similarity enrichment analysis (ChemRICH) was conducted at www.chemrich.fiehnlab.ucdavis.edu [40]. ChemRICH utilizes chemical ontologies and structural similarity to map metabolic modules for interpretation. The input included the annotation name, SMILES code, FDR-corrected *p*-values of Wilcoxon singed rank test, and fold change between the two groups. ChemRICH then uses the Kolmogorov–Smirnov test to calculate significance level (*p*-value) between two diet treatments for all lipid clusters.

### 4.5. GC-TOF MS Analysis for Primary Metabolism

GC-MS analysis for primary metabolites and FFAs were conducted with a previously described method [41]. Briefly, 20 µL rat plasma was extracted with 1 mL of acetonitrile/isopropanol/water (3:3:2). The dried pellets were added with methoxyamine hydrochloride in pyridine for derivatization, followed by N-methyl-N-(trimethylsilyl) trifluoroacetamide (MSTFA, Sigma-Aldrich, St. Louis, MO, USA) for trimethylsilylation. C8–C30 fatty acid methyl esters (FAMEs) were added as internal standard for retention time correction. The derivatized samples were analyzed using a Leco Pegasus IV time of flight mass spectrometer (LECO, St. Joseph, MI, USA) with a Rtx-5Sil MS column (30 m, 0.25 mm i.d., 0.25 microM 95% dimethyl 5% diphenyl polysiloxane film). GC temperature program was 50 °C for 1 min, ramped at 20 °C/min to 330 °C, and then held for 5 min. Electron ionization was at −70 eV. Acquisition was conducted 17 spectra/second with a scan mass range of 85–500 Da. Data processing and metabolite annotation were conducted with BinBase [42]. Raw peak height values were normalized to the sum intensity of all the measured metabolites; in this equation: normalized peak height = Raw peak height/the sum intensity of all measured metabolites in a sample × the averaged intensity of all samples. The technical variance represented by RSD of FAMEs internal standards was 12%.

### 4.6. Other Assays

HOMA-IR was calculated in the basal (i.e., preprandial) states as (glucose [mg/dL] × insulin [μU/mL] ÷ 405 [43]). Plasma glucose was analyzed on a GM9 Glucose Analyzer (Analox Instruments, Stourbridge, UK). Plasma insulin was analyzed using an ELISA kit from ALPCO (Salem, NH, USA; Rat Ultrasensitive Insulin). Total plasma FFA and TG levels were determined as previously described [3].

### 4.7. Statistical Analysis

All data are expressed as means ± S.E.M. The significance of differences in the mean value was assessed by one-way ANOVA followed by post-hoc analysis using the Bonferroni method for multiple comparisons. Metabolomics data were normalized using the sum of all the annotated metabolites. Multivariance analysis and univariance analysis were conducted using MetaboAnalyst 6.0 [44]. Principal component analysis (PCA) was calculated using all annotated lipids (excluding internal standards) from a cube root-transformed, auto-scaled dataset. Heat maps with significantly changed lipids were generated using MetaboAnalyst. Kruskal–Wallis one-way ANOVA followed by post-hoc analysis using Fisher’s least significant difference test were conducted for all the annotated lipids. All *p* values from the large lipidomics dataset were adjusted using Benjamini and Hochberg false discovery rate correction [45]. An adjusted *p* value less than 0.05 was considered statistically significant.

## Figures and Tables

**Table 1:** Food composition and energy intake in the LFD (control) and HFD groups.

	LFD (10% Fat)	HFD (60% Fat)
Food composition (g/kg)		
Casein	210	265
Lard	20	310
Soybean oil	20	30
Corn starch	465	0
Maltodextrin	100	160
Sucrose	90	90
Body weight and energy intake		
Initial body weight (g)	213 ± 2	210 ± 2
Final body weight (g)	316 ± 3	323 ± 4
Energy intake (Kcal/day) week 1	77.2 ± 1.3	88.7 ± 1.5 *
week 2	74.8 ± 1.3	78.1 ± 1.3

Lipid and individual fatty acid contents of the diets are presented in Supplementary Table S2. Values are means ± SEM for 20 rats (body weight) or 10 measurements (energy intake—2 rats per cage). *, *p* < 0.001 vs. LFD.

**Table 2:** Effects of the HFD on plasma levels of individual FFAs in the basal (i.e., preprandial) states. FDR: false discovery rate.

	Fold Change	*p* Value	FDR
FA 16:1 (palmitoleic acid)	0.29	0.00001	0.00011
FA 20:5 (eicosapentaenoic acid)	0.30	0.00004	0.00027
FA 22:6 (docosahexaenoic acid)	0.57	0.00389	0.01152
FA 18:3 (γ-linolenic acid)	0.56	0.00520	0.01497
FA 14:1 (physeteric acid)	0.60	0.00893	0.02376
FA 16:2	0.74	0.02881	0.06344
FA 20:3 (homo-gamma-linolenic acid)	0.71	0.05243	0.10558
FA 24:1	0.89	0.07526	0.14067
FA 20:4 b	1.34	0.12301	0.21000
FA 18:2	0.79	0.16549	0.26751
FA 26:1	0.86	0.16549	0.26751
FA 20:2	1.22	0.21756	0.33250
FA 20:2 (eicosadienoic acid)	1.22	0.21756	0.33250
FA 20:4	0.84	0.21756	0.33250
FA 13:0 (tridecylic acid)	0.93	0.39305	0.51721
FA 15:1	0.85	0.39305	0.51721
FA 17:1	0.85	0.39305	0.51721
FA 19:1	1.16	0.43587	0.55648
FA 18:1	0.93	0.63053	0.73427
FA 22:2 (docosadienoic acid)	0.95	0.68421	0.77572
FA 22:4	1.07	0.68421	0.77572
FA 20:1 (eicosenoic acid)	1.12	0.91180	0.93357

## Data Availability

The data presented in this study are available on the Metabolomics Workbench (https://metabolomicsworkbench.org/; Project doi: http://dx.doi.org/10.21228/M88N7K, accessed 25 July 2024).

## Supplementary Materials

The following supporting information can be downloaded at: https://www.mdpi.com/article/10.3390/ijms25168810/s1.

## Abbreviations

CE: cholesterol ester; CVD: cardiovascular disease; DHA: docosahexaenoic acid; EPA: eicosapentaenoic acid; FFA: free fatty acid; HFD: high-fat diet; HNaLK: high sodium (Na^+^) and low potassium (K^+^); LFD: low-fat diet; PC: phosphatidylcholine; PE: phosphatidylethanolamine; PUFA: polyunsaturated fatty acid; SCD: stearoyl-CoA desaturase; SFA: saturated fatty acid; TG: triglyceride.

## References

[1] Lewis G.F., Hegele R.A. (2022). Effective, disease-modifying, clinical approaches to patients with mild-to-moderate hypertriglyceridaemia. Lancet Diabetes Endocrinol..

[2] Rhee E.P., Cheng S., Larson M.G., Walford G.A., Lewis G.D., McCabe E., Yang E., Farrell L., Fox C.S., O’Donnell C.J. (2011). Lipid profiling identifies a triacylglycerol signature of insulin resistance and improves diabetes prediction in humans. J. Clin. Investig..

[3] Folz J., Oh Y.T., Blaženović I., Richey J., Fiehn O., Youn J.H. (2019). Interaction of gut microbiota and high-sodium, low-potassium diet in altering plasma triglyceride profiles revealed by lipidomics analysis. Mol. Nutr. Food Res..

[4] Schwab U., Seppänen-Laakso T., Yetukuri L., Agren J., Kolehmainen M., Laaksonen D.E., Ruskeepää A.L., Gylling H., Uusitupa M., Oresic M. (2008). Triacylglycerol fatty acid composition in diet-induced weight loss in subjects with abnormal glucose metabolism—The GENOBIN study. PLoS ONE.

[5] Wali J.A., Jarzebska N., Raubenheimer D., Simpson S.J., Rodionov R.N., O’Sullivan J.F. (2020). Cardio-metabolic effects of high-fat diets and their underlying mechanisms-a narrative review. Nutrients.

[6] McDonough A.A., Veiras L.C., Guevara C.A., Ralph D.L. (2017). Cardiovascular benefits associated with higher dietary K+ vs. lower dietary Na+: Evidence from population and mechanistic studies. Am. J. Physiol.-Endo Metab..

[7] Aaron K.J., Sanders P.W. (2013). Role of dietary salt and potassium intake in cardiovascular health and disease: A review of the evidence. Mayo Clin. Proc..

[8] Olga L., van Diepen J.A., Bobeldijk-Pastorova I., Gross G., Prentice P.M., Snowden S.G., Furse S., Kooistra T., Hughes I.A., Schoemaker M.H. (2021). Lipid ratios representing SCD1, FADS1, and FADS2 activities as candidate biomarkers of early growth and adiposity. EBioMedicine.

[9] Shaikh S.R., Virk R., Van Dyke T.E. (2022). Potential mechanisms by which hydroxyeicosapentaenoic acids regulate glucose homeostasis in obesity. Adv. Nutr..

[10] Jiang S., Han S., Wang D.W. (2024). The involvement of soluble epoxide hydrolase in the development of cardiovascular diseases through epoxyeicosatrienoic acids. Front. Pharmacol..

[11] Ortiz M., Soto-Alarcón S.A., Orellana P., Espinosa A., Campos C., López-Arana S., Rincón M.A., Illesca P., Valenzuela R., Videla L.A. (2020). Suppression of high-fat diet-induced obesity-associated liver mitochondrial dysfunction by docosahexaenoic acid and hydroxytyrosol co-administration. Dig. Liver Dis..

[12] Illesca P., Valenzuela R., Espinosa A., Echeverría F., Soto-Alarcon S., Campos C., Rodriguez A., Vargas R., Magrone T., Videla L.A. (2020). Protective effects of eicosapentaenoic acid plus hydroxytyrosol supplementation against white adipose tissue abnormalities in mice fed a high-fat diet. Molecules.

[13] Valenzuela R., Espinosa A., González-Mañán D., D’Espessailles A., Fernández V., Videla L.A., Tapia G. (2012). N-3 long-chain polyunsaturated fatty acid supplementation significantly reduces liver oxidative stress in high fat induced steatosis. PLoS ONE.

[14] Chu K.Y., Mellet N., Thai L.M., Meikle P.J., Biden T.J. (2020). Short-term inhibition of autophagy benefits pancreatic β-cells by augmenting ether lipids and peroxisomal function, and by countering depletion of n-3 polyunsaturated fatty acids after fat-feeding. Mol. Metab..

[15] Matikainen N., Adiels M., Söderlund S., Stennabb S., Ahola T., Hakkarainen A., Borén J., Taskinen M.R. (2014). Hepatic lipogenesis and a marker of hepatic lipid oxidation, predict postprandial responses of triglyceride-rich lipoproteins. Obesity (Silver Spring).

[16] Biddinger S.B., Almind K., Miyazaki M., Kokkotou E., Ntambi J.M., Ronald K. (2005). Effects of diet and genetic background on sterol regulatory element-binding protein-1c, stearoyl-CoA desaturase 1, and the development of the metabolic syndrome. Diabetes.

[17] Ampong I., John Ikwuobe O., Brown J.E.P., Bailey C.J., Gao D., Gutierrez-Merino J., Griffiths H.R. (2022). Odd chain fatty acid metabolism in mice after a high fat diet. Int. J. Biochem. Cell Biol..

[18] Gianotti T.F., Burgueño A., Mansilla N.G., Pirola C.J., Sookoian S. (2013). Fatty liver is associated with transcriptional downregulation of stearoyl-CoA desaturase and impaired protein dimerization. PLoS ONE.

[19] Warensjo E., Riserus U., Gustafsson I.B., Mohsen R., Cederholm T., Vessby B. (2008). Effects of saturated and unsaturated fatty acids on estimated desaturase activities during a controlled dietary intervention. Nutr. Metab. Cardiovasc. Dis..

[20] Ntambi J.M., Miyazaki M., Stoehr J.P., Lan H., Kendziorski C.M., Yandell B.S., Song Y., Cohen P., Friedman J.M., Attie A.D. (2002). Loss of stearoyl-CoA desaturase-1 function protects mice against adiposity. Proc. Natl. Acad. Sci. USA.

[21] Miyazaki M., Kim Y.C., Gray-Keller M.P., Attie A.D., Ntambi J.M. (2000). The biosynthesis of hepatic cholesterol esters and triglycerides is impaired in mice with a disruption of the gene for stearoyl-CoA desaturase 1. J. Biol. Chem..

[22] Miyazaki M., Flowers M.T., Sampath H., Chu K., Otzelberger C., Liu X., Ntambi J.M. (2007). Hepatic stearoyl-CoA desaturase-1 deficiency protects mice from carbohydrate-induced adiposity and hepatic steatosis. Cell Metab..

[23] Miyazaki M., Sampath H., Liu X., Flowers M.T., Chu K., Dobrzyn A., Ntambi J.M. (2009). Stearoyl-CoA desaturase-1 deficiency attenuates obesity and insulin resistance in leptin-resistant obese mice. Biochem. Biophys. Res. Commun..

[24] Cao H., Gerhold K., Mayers J.R., Wiest M.M., Watkins S.M., Hotamisligil G.S. (2008). Identification of a lipokine, a lipid hormone linking adipose tissue to systemic metabolism. Cell.

[25] Stefan N., Kantartzis K., Celebi N., Staiger H., Machann J., Schick F., Cegan A., Elcnerova M., Schleicher E., Fritsche A. (2010). Circulating palmitoleate strongly and independently predicts insulin sensitivity in humans. Diabetes Care.

[26] Fabbrini E., Magkos F., Su X., Abumrad N.A., Nejedly N., Coughlin C.C., Okunade A.L., Patterson B.W., Klein S. (2011). Insulin sensitivity is not associated with palmitoleate availability in obese humans. J. Lipid. Res..

[27] Abbas A.M., Sakr H.F. (2013). Simvastatin and vitamin E effects on cardiac and hepatic oxidative stress in rats fed on high fat diet. J. Physiol. Biochem..

[28] Sarna L.K., Wang N.W.P., Hwang S., Siow Y.L., Karmin O. (2012). Folic acid supplementation attenuates high fat diet induced hepatic oxidative stress via regulation of NADPH oxidase. Can. J. Physiol. Pharmacol..

[29] Dean J.M., Lodhi I.J. (2018). Structural and functional roles of ether lipids. Protein Cell.

[30] Goodenowe D.B., Haroon J., Kling M.A., Zielinski M., Mahdavi K., Habelhah B., Shtilkind L., Jordan S. (2022). Targeted Plasmalogen Supplementation: Effects on Blood Plasmalogens, Oxidative Stress Biomarkers, Cognition, and Mobility in Cognitively Impaired Persons. Front. Cell Dev. Biol..

[31] Engelmann B. (2004). Plasmalogens: Targets for oxidants and major lipophilic antioxidants. Biochem. Soc. Trans..

[32] Ferdinandusse S., McWalter K., Te Brinke H., IJlst L., Mooijer P.M., Ruiter J.P.N., van Lint A.E.M., Pras-Raves M., Wever E., Millan F. (2021). An autosomal dominant neurological disorder caused by de novo variants in FAR1 resulting in uncontrolled synthesis of ether lipids. Genet. Med..

[33] Jia R., Cao L.-P., Du J.-L., He Q., Gu Z.-Y., Jeney G., Xu P., Yin G.-J. (2020). Effects of high-fat diet on antioxidative status, apoptosis and inflammation in liver of tilapia (*Oreochromis niloticus*) via Nrf2, TLRs and JNK pathways. Fish Shellfish Immunol..

[34] Dai F., Jiang T., Bao Y.-Y., Chen G.-J., Chen L., Zhang Q., Lu Y.-X. (2016). Fenofibrate improves high-fat diet-induced and palmitate-induced endoplasmic reticulum stress and inflammation in skeletal muscle. Life Sci..

[35] Cajka T., Smilowitz J.T., Fiehn O. (2017). Validating quantitative untargeted lipidomics across nine liquid chromatography-high-resolution mass spectrometry platforms. Anal. Chem..

[36] Matyash V., Liebisch G., Kurzchalia T.V., Shevchenko A., Schwudke D. (2008). Lipid extraction by methyl-tert-butyl ether for high-throughput lipidomics. J. Lipid Res..

[37] Koelmel J.P., Kroeger N.M., Gill E.L., Ulmer C.Z., Bowden J.A., Patterson R.E., Yost R.A., Garrett T.J. (2017). Expanding lipidome coverage using LC-MS/MS data-dependent acquisition with automated exclusion list generation. J. Am. Soc. Mass Spectrom..

[38] Tsugawa H., Ikeda K., Takahashi M., Satoh A., Mori Y., Uchino H., Okahashi N., Yamada Y., Tada I., Bonini P. (2020). A lipidome atlas in MS-DIAL 4. Nat. Biotechnol..

[39] Defelice B.C., Mehta S.S., Samra S., Wancewicz B., Fahrmann J.F., Fiehn O. (2017). Mass Spectral Feature List Optimizer (MS-FLO): A tool to minimize false positive peak reports in untargeted liquid chromatography-mass spectroscopy (LC-MS) data processing. Anal. Chem..

[40] Barupal D.K., Fiehn O. (2017). Chemical Similarity Enrichment Analysis (ChemRICH) as alternative to biochemical pathway mapping for metabolomic data sets. Sci. Rep..

[41] Barupal D.K., Zhang Y., Shen T., Fan S., Roberts B.S., Fitzgerald P., Wancewicz B., Valdiviez L., Wohlgemuth G., Byram G. (2019). A comprehensive plasma metabolomics dataset for a cohort of mouse knockouts within the international mouse phenotyping consortium. Metabolites.

[42] Skogerson K., Wohlgemuth G., Barupal D.K., Fiehn O. (2011). The volatile compound BinBase mass spectral database. BMC Bioinform..

[43] Matthews D.R., Hosker J.P., Rudenski A.S., Naylor B.A., Treacher D.F., Turner R.C. (1985). Homeostasis model assessment: Insulin resistance and beta-cell function from fasting plasma glucose and insulin concentrations in man. Diabetologia.

[44] Benjamini Y., Hochberg Y. (1995). Controlling the false discovery rate: A practical and powerful approach to multiple testing. J. R. Stat. Soc. Ser. B.

[45] Chong J., Soufan O., Li C., Caraus I., Li S., Bourque G., Wishart D.S., Xia J. (2018). MetaboAnalyst 4.0: Towards more transparent and integrative metabolomics analysis. Nucleic Acids Res..

