# Supplementary material for: High-Fat Feeding Alters Circulating Triglyceride Composition: Roles of FFA Desaturation and ω-3 Fatty Acid Availability
Source: Int J Mol Sci. 2024 Aug 13;25(16):8810. doi: 10.3390/ijms25168810 (PMC11354557; doi:10.3390/ijms25168810)
Supplement: Supplementary file 1 [file ijms-25-08810-s001.zip › Supplemental Figures.pdf]

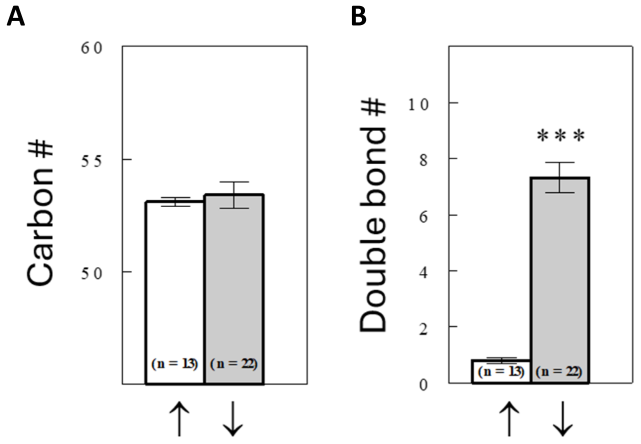

Supplemental Figure S1. Carbon and double bond numbers of TGs significantly increased (↑) or decreased (↓) by more than 2-fold by the HFD. Data are means ± SEM (*n* = 13 or 22). ***, P < 0.001 vs. ↑.

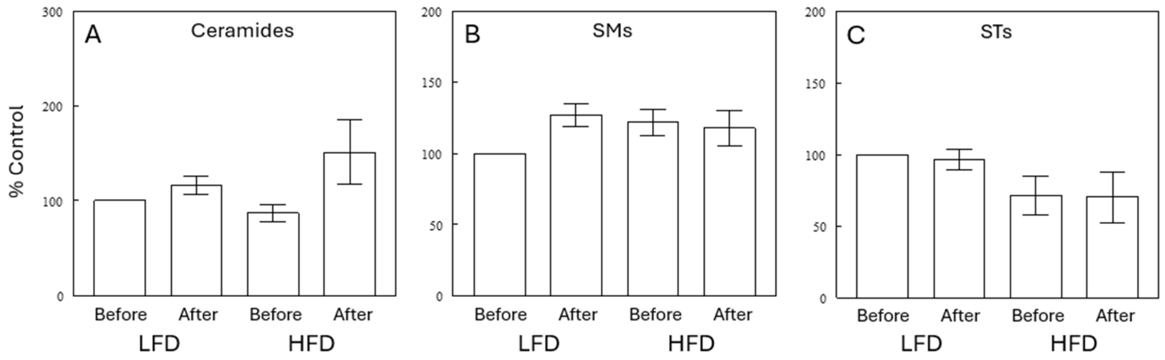

Supplemental Figure S2. Effects of diets on circulating, oxidized FA-containing ceramides (***A***), SMs (***B***), and STs (***C***) in the preprandial (Before Meal) and postprandial (After Meal) states. Data are means ± SEM (*n* = 17 for ceramides, 29 for SMs, and 4 for STs).

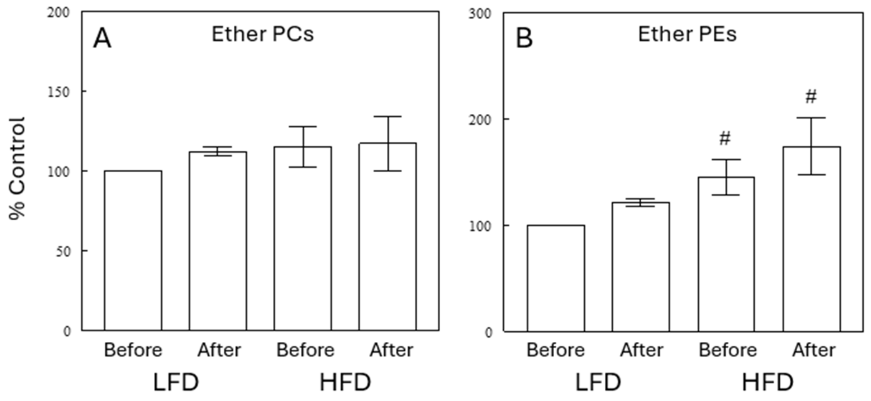

Supplemental Figure S3. Effects of diets on ether PCs (***A***) and ether PEs (***B***) in the preprandial (Before Meal) and postprandial (After Meal) states. Data are means ± SEM (*n* = 22 for ether PCs and 24 for ether PEs). #, P < 0.05 vs. LFD.

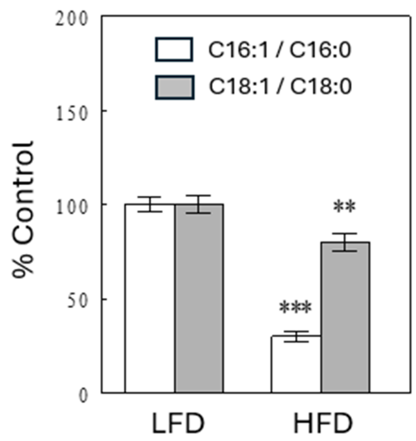

Supplemental Figure S4. SCD activity indices estimated from plasma FFAs as the ratio of C16:1 to C16:0 or C18:1 to C18:0. Data are means ± SEM (n = 10). **, p < 0.01; ***, p < 0.001 vs. LFD.
