# Supplementary material for: High-Fat Feeding Alters Circulating Triglyceride Composition: Roles of FFA Desaturation and ω-3 Fatty Acid Availability
Source: Int J Mol Sci. 2024 Aug 13;25(16):8810. doi: 10.3390/ijms25168810 (PMC11354557; doi:10.3390/ijms25168810)
Supplement: Supplementary file 1 [file ijms-25-08810-s001.zip › Supplemental Table S2.pdf]

Supplemental Table S2. Lipid and fatty acid contents of the HFD and LFD.

	HFD (TD.06414)	LFD (TD.08806)
**Fatty Acids**		
Total Fat (g/kg)	342.7	42.1
Saturated Fat (g/kg)	119.2	10.4
Monounsaturated Fat (g/kg)	134.0	12.8
Polyunsaturated Fat (g/kg)	77.2	16.0
Saturated Fat (%)	36.1	26.5
Monounsaturated Fat (%)	40.6	32.7
Polyunsaturated Fat (%)	23.4	40.8
4:0 Butyric (g/kg)	0.0	0.0
6:0 Caproic (g/kg)	0.0	0.0
8:0 Caprylic (g/kg)	0.0	0.0
10:0 Capric (g/kg)	0.0	0.0
12:0 Lauric (g/kg)	0.0	0.0
14:0 Myristic (g/kg)	3.1	0.2
16:0 Palmitic (g/kg)	74.6	6.8
16:1 Palmitoleic (g/kg)	6.2	0.4
18:0 Stearic (g/kg)	41.5	3.4
18:1 Oleic (g/kg)	127.8	12.4
18:2 Linoleic (g/kg)	71.7	14.2
18:3 Linolenic (g/kg)	5.5	1.8
**ω-3: ω-6 ratio**	0.08	0.13
**Cholesterol (mg/kg)**	347.5	61.0

Values, calculated from data of included ingredients, were provided by the vendor.
